# Phage Therapy of *Mycobacterium* Infections: Compassionate Use of Phages in 20 Patients With Drug-Resistant Mycobacterial Disease

**DOI:** 10.1093/cid/ciac453

**Published:** 2022-06-09

**Authors:** Rebekah M Dedrick, Bailey E Smith, Madison Cristinziano, Krista G Freeman, Deborah Jacobs-Sera, Yvonne Belessis, A Whitney Brown, Keira A Cohen, Rebecca M Davidson, David van Duin, Andrew Gainey, Cristina Berastegui Garcia, C R Robert George, Ghady Haidar, Winnie Ip, Jonathan Iredell, Ameneh Khatami, Jessica S Little, Kirsi Malmivaara, Brendan J McMullan, David E Michalik, Andrea Moscatelli, Jerry A Nick, Maria G Tupayachi Ortiz, Hari M Polenakovik, Paul D Robinson, Mikael Skurnik, Daniel A Solomon, James Soothill, Helen Spencer, Peter Wark, Austen Worth, Robert T Schooley, Constance A Benson, Graham F Hatfull

**Affiliations:** Department of Biological Sciences, University of Pittsburgh, Pittsburgh, Pennsylvania, USA; Department of Biological Sciences, University of Pittsburgh, Pittsburgh, Pennsylvania, USA; Department of Biological Sciences, University of Pittsburgh, Pittsburgh, Pennsylvania, USA; Department of Biological Sciences, University of Pittsburgh, Pittsburgh, Pennsylvania, USA; Department of Biological Sciences, University of Pittsburgh, Pittsburgh, Pennsylvania, USA; School of Women's and Children's Health, University of New South Wales, Sydney, New South Wales, Australia; Department of Respiratory Medicine, Sydney Children's Hospital, Sydney, New South Wales, Australia; Inova Fairfax Hospital, Falls Church, Virginia, USA; Division of Pulmonary and Critical Care Medicine, Johns Hopkins University School of Medicine, Baltimore, Maryland, USA; Center for Genes, Environment, and Health, National Jewish Health, Denver, Colorado, USA; Division of Infectious Diseases, University of North Carolina, Chapel Hill, North Carolina, USA; Department of Pharmacy, Division of Pediatric Infectious Diseases, Prisma Health Children's Hospital–Midlands, Columbia, South Carolina, USA; Department of Respiratory Disease, Hospital Universitari Vall d’Hebron, Barcelona, Spain; New South Wales Health Pathology Microbiology, John Hunter Hospital, New Lambton, New South Wales, Australia; Department of Medicine, Division of Infectious Diseases, University of Pittsburgh School of Medicine, Pittsburgh, Pennsylvania, USA; Department of Pediatric Immunology, Great Ormond Street Hospital, London, United Kingdom; Department of Immunology and Infectious Diseases, Sydney Children’s Hospital, Randwick, New South Wales, Australia; Department of Infectious Diseases and Microbiology, Children's Hospital at Westmead, Westmead, New South Wales, Australia; Discipline of Child and Adolescent Health, University of Syndey, Sydney, New South Wales, Australia; Division of Infectious Diseases, Brigham and Women’s Hospital, Boston, Massachusetts, USA; Great Ormond Street Hospital, London, United Kingdom; Department of Immunology and Infectious Diseases, Sydney Children’s Hospital, Randwick, New South Wales, Australia; Miller Children’s and Women’s Hospital, Division of Pediatric Infectious Diseases, Long Beach, California, USA; Neonatal and Pediatric Intensive Care Unit, Instituto Giannina Gaslini, Genoa, Italy; Department of Medicine, National Jewish Health, Denver, Colorado, USA; Department of Medicine, Division of Pulmonary and Critical Care Medicine, Miller School of Medicine, University of Miami, Miami, Florida, USA; Internal Medicine Department, Dayton Children’s Hospital, Boonshoft School of Medicine, Wright State University, Dayton, Ohio, USA; Department of Respiratory Medicine, The Children's Hospital at Westmead, Westmead, New South Wales, Australia; Department of Bacteriology and Immunology, Human Microbiome Research Program, University of Helsinki, Helsinki, Finland; Division of Clinical Microbiology, Helsinki University Hospital, Helsinki, Finland; Division of Infectious Diseases, Brigham and Women’s Hospital, Boston, Massachusetts, USA; Great Ormond Street Hospital, London, United Kingdom; Respiratory Medicine and Cardiothoracic Transplantation, Great Ormond Street Hospital, London, United Kingdom; Immune Health Program, Hunter Medical Research Institute, University of Newcastle, Callaghan, New South Wales, Australia; Department of Pediatric Immunology, Great Ormond Street Hospital, London, United Kingdom; Division of Infectious Diseases and Global Public Health, Department of Medicine, University of California, San Diego, San Diego, California, USA; Division of Infectious Diseases and Global Public Health, Department of Medicine, University of California, San Diego, San Diego, California, USA; Department of Biological Sciences, University of Pittsburgh, Pittsburgh, Pennsylvania, USA

**Keywords:** phage therapy, nontuberculous mycobacteria, mycobacteriophage

## Abstract

**Background:**

Nontuberculous *Mycobacterium* infections, particularly *Mycobacterium abscessus*, are increasingly common among patients with cystic fibrosis and chronic bronchiectatic lung diseases. Treatment is challenging due to intrinsic antibiotic resistance. Bacteriophage therapy represents a potentially novel approach. Relatively few active lytic phages are available and there is great variation in phage susceptibilities among *M. abscessus* isolates, requiring personalized phage identification.

**Methods:**

*Mycobacterium* isolates from 200 culture-positive patients with symptomatic disease were screened for phage susceptibilities. One or more lytic phages were identified for 55 isolates. Phages were administered intravenously, by aerosolization, or both to 20 patients on a compassionate use basis and patients were monitored for adverse reactions, clinical and microbiologic responses, the emergence of phage resistance, and phage neutralization in serum, sputum, or bronchoalveolar lavage fluid.

**Results:**

No adverse reactions attributed to therapy were seen in any patient regardless of the pathogen, phages administered, or the route of delivery. Favorable clinical or microbiological responses were observed in 11 patients. Neutralizing antibodies were identified in serum after initiation of phage delivery intravenously in 8 patients, potentially contributing to lack of treatment response in 4 cases, but were not consistently associated with unfavorable responses in others. Eleven patients were treated with only a single phage, and no phage resistance was observed in any of these.

**Conclusions:**

Phage treatment of *Mycobacterium* infections is challenging due to the limited repertoire of therapeutically useful phages, but favorable clinical outcomes in patients lacking any other treatment options support continued development of adjunctive phage therapy for some mycobacterial infections.

## Teaser

We describe 20 consecutive cases of bacteriophage treatment of *Mycobacterium* infections. We observed no adverse reactions, favorable outcomes in at least 50% of patients, no evidence of phage resistance, and neutralizing immune reactions that do not correlate with treatment success.

The therapeutic use of phages for treating drug-resistant bacterial infections has received recent attention, but the types of infections and pathogens deemed suitable; routes, dosage, and frequency of administration; interactions with antibiotics; and pharmacokinetics remain unclear [1, 2]. Unlike small molecule antibiotics, bacteriophages can replicate at the sites of infection, are much larger than standard antimicrobials, and penetration to sites of bacterial replication may be restricted. Immune neutralization may also limit phage activity [3]. Bacteriophages are often highly bacterium-specific, which is advantageous for precise pathogen targeting, but demands personalized phage matching for individual patient isolates [3]. Anecdotal reports support a robust safety profile and clinical improvement has been reported for some but not all cases [4–7].

Because of increasing and widespread antibiotic resistance among *Mycobacterium* pathogens, alternative therapies are needed [8–10]. Nontuberculous mycobacterial (NTM) infections—especially those caused by *Mycobacterium abscessus*—are particularly challenging, as many are refractory to antibiotics and extended drug therapies are poorly tolerated [11]. NTM infections are increasingly common among people with cystic fibrosis (CF), but are also prevalent among non-CF patients, including those with bronchiectasis or Mendelian susceptibility to mycobacterial disease (MSMD) [12, 13]. People with CF typically have complex recurrent pulmonary infections, often with mixed flora, but *M. abscessus* infections are particularly challenging and typically preclude lung transplantation. Phages have been proposed for managing CF [14], but their utility for *Mycobacterium* infections, including NTM and tuberculosis [3, 15, 16], remains unclear.

There is great variation in phage susceptibilities among *M. abscessus* clinical isolates [17]. Approximately 40% of *M. abscessus* isolates have a smooth colony morphotype [17, 18], and to date no therapeutically useful phages have been identified for these [17]. In contrast, 75%–80% of rough strains are efficiently killed by at least 1 phage, and the low rates of phage resistance in vitro suggest that phage resistance in vivo may not be a limitation [17]. Nonetheless, the repertoire of therapeutically useful phages is small, and mostly limited to phages isolated on *Mycobacterium smegmatis*; few phages have been isolated directly on any strain of *M. abscessus* [5].

Two case reports have described compassionate use of phages for NTM infections [5, 19]. One was a patient with CF and disseminated *M. abscessus* infection following bilateral lung transplantation and drug-induced immunosuppression [5]. The second was an immunocompetent patient with non-CF bronchiectasis and a severe *M. abscessus* pulmonary infection [19]. The same 3-phage cocktail was used to treat both patients and was administered intravenously (IV) twice daily at a dose of 10^9^ plaque-forming units (PFUs) per dose for at least 6 months. Both were also treated with concomitant multidrug antibiotic regimens. The first patient had clinical improvement in lung function, radiographic imaging, and clinical signs and symptoms, although without complete clearance of the infection [5]. The second had initial reduction in *M. abscessus* colony counts in sputum that was abrogated by emergence of a potent neutralizing antibody response to the phages [19].

We report here therapeutic interventions for a pilot cohort of 20 patients with antibiotic-refractory mycobacterial infections. Phage administration was safe, no resistance was observed, and favorable microbiological or clinical outcomes were observed in a majority of cases.

## METHODS

### Identification and Evaluation of Patients Suitable for Compassionate Use Phage Treatment

Since May 2019, we received approximately 200 requests for adjunctive phage treatment for patients with NTM infections that were either refractory to antibiotic therapies or in which extended drug treatments were not tolerated; antibiotic susceptibilities are shown in Supplementary Table 1. Most patients had CF and *M. abscessus* infections, but some had other underlying diseases complicated by NTM (and in 1 case bacille Calmette-Guerin [BCG]) infections. The following criteria determined eligibility for compassionate use phage therapy: age >5 years, microbiologic documentation of mycobacterial infection based on at least 2 positive cultures of relevant tissue or body fluids; drug susceptibility testing (DST) documenting resistance to multiple antimycobacterial drugs; clinical signs, symptoms and radiographic findings involving at least 1 organ system (eg, NTM lung disease based on American Thoracic Society (ATS), European Respiratory Society (ERS), European Society of Clinical Microbiology and Infectious Diseases (ESCMID), and Infectious Diseases Society of America (IDSA) diagnostic criteria [20]) or evidence of disseminated disease; clinical failure of or intolerance to antimycobacterial treatment; stable underlying conditions with anticipated survival of at least 3 months; and treating physician(s) able and willing to use adjunctive phage therapy. Recent mycobacterial isolates from patients were screened for phages that efficiently infect and kill the isolate, and regulatory approval was provided under an emergency Investigational New Drug application by the United States Food and Drug Administration or comparable processes outside of the United States. Local investigational or ethics review board approvals were also obtained. Twenty patients who met criteria provided informed consent for adjunctive phage therapy (Table 1). See the Supplementary Information for detailed methods.

**Table 1.** Profiles of 20 Patients Treated for *Mycobacterium* Infections With Phages

Patient No., Origin, Strain	Age	Underlying Condition	Type of Infection	Organism	Phages Used	Route of Administration (Duration)	Key Clinical Data and Outcomes
Favorable or partial responses							
1, UK GD01	Pediatric	CF, bilateral lung transplant	Disseminated (lung, skin and liver nodules, sternal bone infection)	*Mycobacterium abscessus* subsp *massiliense*	BPsΔ*33*HTH_HRM10, Muddy, ZoeJΔ*45*	IV, topical (3.5 y)	Resolution of infected liver and skin nodules, sternal wound closure; intermittent smear and culture negative; patient died due to multiple transplant complications
2, USA GD10	Adult	Scleroderma, lung transplant	Lung, sternal bone infection	*M. abscessus* subsp *massiliense*	Muddy	IV, chest wash (1 m)	Swab samples became AFB smear negative/rare; patient died due to multiple underlying coinfections
4, Italy GD24	Pediatric	CF, lung transplant	Lung	*M. abscessus* subsp *abscessus*	BPsΔ*33*HTH_HRM^GD03^	IV (1 m)	Conversion to AFB smear negative; patient died due to adenovirus systemic infection
7, Australia GD43	Pediatric	CF	Lung	*M. abscessus* subsp *abscessus* (both smooth and rough colonies isolated; only rough colony morphotype susceptible to phage)	BPsΔ*33*HTH_HRM^GD03^	IV (7 m)	Culture conversion to negative for rough colony strain, but smooth strain still present; improvement in some clinical signs and symptoms
9, Australia GD54	Pediatric	CF	Lung	*M. abscessus* subsp *abscessus*	Muddy, BPsΔ*33*HTH_HRM^GD03^	IV, Bronchoscopic administration (1 y)	After 10 mo of IV therapy and 2 phage bronchoscopic administrations, patient was AFB smear and culture negative; infection resolved
10, Spain GD57	Adult	CF, lung transplant	Lung, disseminated infection post–lung transplant	*M. abscessus* subsp *abscessus*	BPsΔ*33*HTH_HRM^GD03^, Itos	IV (1 y)	Culture and smear conversion; improved FEV_1_ and resolution of infection
13, Finland GD102	Adult	CF	Lung	*M. abscessus* subsp *abscessus*	Muddy	Course 1: IV (10 m) Course 2: Aerosol (4 wk)	Patient had FEV_1_ improvement but remained AFB culture positive; switched to aerosolized phage therapy
15, USA GD116	Adult	CF	Lung	*M. abscessus* subsp *abscessus*	BPsΔ*33*HTH_HRM10, D29_HRM^GD40^	IV (1.1 y, ongoing)	After 12 mo of IV phage therapy, culture negative and eligible for lung transplant. Patient was transplanted October 2021; no recurrence
16, USA GD153	Adult	Seronegative arthritis on immunosuppression	Disseminated cutaneous infection	*Mycobacterium chelonae*	Muddy	IV (9 m, ongoing)	Skin nodules significantly improved and tissue culture and histopathology negative for organism after 3 mo of IV phage
17, USA GD156	Pediatric	CF	Lung	*Mycobacterium avium* complex	Muddy	Course 1: IV (3 m) Course 2: Aerosol (ongoing)	FEV_1_ improvement of >15% was seen in first month of IV phage treatment; time to positivity increased from 1 wk prior to treatment to 4–8 wk posttreatment; consecutive AFB cultures obtained at 21 and 25 wk after treatment have shown no growth
20, UK BCG	Pediatric	Heterozygous mutation in *NFKBIA* gene c.32 G > A; p.(Trp11*), MSMD	Disseminated BCG mycobacterial infection	BCG	Muddy, D29, FionnbharthΔ*43*Δ*45*, Fred313cpmΔ*33*	IV (1.7 y)	Reduction in fever and systemic inflammation; improved BCG PCR cycle threshold in cultures. Patient died from other infections
Complex, inconclusive, or incomplete responses							
3, USA GD20	Adult	CF	Lung	*M. abscessus* subsp *abscessus*	BPsΔ*33*HTH_HRM10, Itos	IV (both phages, 2 d) Break 18 d IV (BPsΔ*33*HTH_HRM10 only, 8 d)	Patient died due to multiple organ failure
8, USA GD45	Adult	CF	Lung	*M. abscessus* subsp *abscessus*	Muddy	Course 1: Aerosol (3 m) Course 2: IV + Aerosol at 10^10^ PFU/dose (7 m) Course 3: Aerosol at 10^10^ PFU/dose (3 m) Course 4: Aerosol Muddy and BPs (2 m, ongoing)	No clinical improvement with aerosolized phage, IV delivery added, and patient became intermittently smear negative; patient mounted a neutralizing antibody response, although was intermittently smear negative. Patient was switched to aerosolized phage only
11, USA GD68	Pediatric	CF	Lung	*M. abscessus* subsp *massiliense* (both smooth and rough colony morphologies; only rough was treated with phage)	Muddy	IV (1.7 y, ongoing)	Improved chest scans, but intermittently AFB smear and culture positive
12, USA GD82	Adult	Chronic lung bronchiectasis	Lung	*M. abscessus* subsp *massiliense*	BPsΔ*33*HTH_HRM10, Muddy, ZoeJΔ*45*	Course 1: IV (6 m) Course 2: Aerosol (12 m, discontinued in terminal illness)	Little clinical improvement with IV phage therapy; potent phage neutralization; switched to aerosolized phage; phage therapy continued through terminal illness; patient died in hospice
18, USA GD158	Adult	CF	Lung	*M. abscessus* subsp *massiliense*	Muddy	Course 1: IV (4 m) Course 2: IV and aerosol (ongoing)	Little clinical improvement with IV phage therapy; potent phage neutralization; added aerosolized phage; treatment ongoing
No evident clinical improvement							
5, USA GD25	Adult	CF	Lung	*M. abscessus* subsp *abscessus*	Muddy	Course 1: IV (3 m) Break 2 m Course 2: Aerosol (8 m)	No substantial clinical improvement after IV or aerosolized treatment
6, Australia GD40	Adult	CF	Lung	*M. abscessus* subsp *abscessus*	BPsΔ*33*HTH_HRM^GD03^	IV (6 m)	No substantial clinical improvement after 6 mo IV treatment
14, Spain GD113	Adult	CF	Lung	*M. abscessus* subsp *abscessus*	BPsΔ*33*HTH_HRM^GD03^, D29_HRM^GD40^	IV (11 m, ongoing)	No clinical improvement after 11 mo
19, USA GD194	Adult	CF	Lung	*M. abscessus* subsp *abscessus*	BPsΔ*33*HTH_HRM10, Itos	Course 1: IV (5 m) Course 2: Aerosol (<1 m, ongoing)	No substantial clinical improvement after 3 months of IV phage. Patient was switched to aerosolized phage treatment; ongoing

Abbreviations: AFB, acid-fast bacilli; BCG, bacille Calmette-Guerin; CF, cystic fibrosis; FEV_1_, forced expiratory volume at 1 second; IV, intravenous; MSMD, Mendelian susceptibility to mycobacterial disease; PCR, polymerase chain reaction; PFU, plaque-forming units; UK, United Kingdom; USA, United States of America.

### Phage Administration

With relatively few potentially therapeutically useful phages for NTM [17], in 11 cases only a single candidate phage was identified; for others, 2 or more genomically distinct phages were combined into cocktails (Tables 1 and 2). Most patients were initially administered 10^9^ PFU intravenously twice daily; some patients also received the same dose by inhalational nebulization (Table 1). All patients also received antimycobacterial treatment with at least 2 drugs based on prior DST against their own target isolate and on their prior tolerance of available drugs. Initial duration of phage treatment was 6 months, although some patients received shorter or longer courses of treatment directed by clinical and microbiologic responses (Table 1). When available, baseline clinical and laboratory assessments of patients included signs and symptoms of NTM infection, a complete blood count, renal and liver chemistries, sedimentation rate, and C-reactive protein, and, when applicable, radiographic assessment and pulmonary function studies. Where possible, these were monitored weekly during the first month of phage treatment and monthly thereafter; radiographic and pulmonary functions were evaluated at intervals determined by treating clinicians. Microbiological monitoring included acid-fast bacilli (AFB) smear and culture of sputum or other relevant clinical specimens at baseline, at 1 month, and then periodically thereafter based on clinical and microbiologic response. During treatment, *Mycobacterium* strains from clinical samples were tested for phage resistance (Table 2). Where possible, serum, sputum, and/or bronchoalveolar lavage specimens were collected prior to and after treatment initiation and tested for phage antibodies using neutralization or enzyme-linked immunosorbent assays or both (Table 3). For several patients, both serum and sputum samples were tested by polymerase chain reaction (PCR) for the presence of phage DNA; only sporadic weakly positive signals were observed (Supplementary Figure 1). Antimycobacterial regimens were adjusted by the treating clinicians as needed based on DST and drug tolerability during phage treatment.

**Table 2.** Therapeutic Phages and Phage Sensitivities of Posttreatment *Mycobacterium* Isolates

Phages^a^	No. of Patients Treated	Phage Susceptibility of Phage Treatment Isolates^b,c^
Muddy	8	S – Patients 2, 5, 8, 11 (smooth and rough)^d^, 13, 18 NA – Patient 16 NT – Patient 17
BPsΔ*33*HTH_HRM^GD03^	3	Only non-susceptible smooth strain recovered post treatment – Patient 7^d^ NT – Patients 4, 6
Muddy, BPsΔ*33*HTH_HRM^GD03^	1	NA – Patient 9
Muddy, BPsΔ*33*HTH _HRM10, ZoeJΔ*45*	2	S (BPsΔ*33*HTH_HRM10 and Muddy) – Patients 1, 12; PR (ZoeJΔ*45*) – Patients 1, 12
BPsΔ*33*HTH_HRM^GD03^, Itos	1	NT – Patient 10
BPsΔ*33*HTH_HRM10, Itos	2	S – Patient 19 NT – Patient 3
BPsΔ*33*HTH_HRM10, D29_HRM^GD40^	1	S – Patient 15
BPsΔ*33*HTH_HRM^GD03^, D29_HRM^GD40^	1	NT – Patient 14
Muddy, D29, FionnbharthΔ*43*Δ*45*, Fred313cpmΔ*33*	2	NT – Patient 20

Abbreviations: NA, not applicable (no posttherapy isolates were grown); NT, not tested; PR, partially resistant; S, sensitive.

Phages were used therapeutically either singly, or in the combinations shown.

Patient numbers are as shown in Tables 1 and 3.

Strains from patients were tested for changes in phage sensitivity. Strains were initially sensitive to the phages used therapeutically. For each patient, strains isolated after the start of phage treatment are shown as being phage sensitive, partially resistant, or not tested. Multiple samples from the same patients were tested similarly.

Indicates patient with a mixed smooth and rough colony morphology *Mycobacterium abscessus* infection. For patient 11, both smooth and rough morphotypes were recovered during treatment and both morphotypes remained fully susceptible to Muddy, although the smooth strain is not killed by Muddy. For patient 7, only smooth isolates were found intratreatment, and only smooth isolates were tested for phage susceptibility.

**Table 3.** Serum and Sputum Phage Neutralization

Patient^a^	Strain	Immune Status	Sample	Phage	Neutralization^b^	Pre-phage ELISA Log Half-Maximum Titer^c^	Maximum Observed ELISA Log Half-Maximum Titer^c^
1	GD01	IS	Serum	Muddy	Y (2.3 y)	NA	NA
				BPsΔ33HTH_HRM10	N	NA	NA
				ZoeJΔ45	N	NA	NA
3	GD20	IC	Serum	BPsΔ*33*HTH_HRM10	N	2.8	3.5 (24 h)
				Itos	N	NA	NA
			Sputum	BPsΔ*33*HTH_HRM10	N	1.8	3.4 (3 wk)
				Itos	N	NA	NA
5	GD25	IC	Serum	Muddy	N	NA	NA
7	GD43	IC	Serum	BPsΔ*33*HTH_HRM^GD03^	N	NA	NA
			BAL	BPsΔ*33*HTH_HRM^GD03^	N	NA	NA
8	GD45	IC	Serum	Muddy	Y (1 m)	4.3	4.6 (1 m IV)
			Sputum	Muddy	N	NA	1.7 (28 d)
9	GD54	IC	Serum	Muddy	N	NA	NA
				BPsΔ*33*HTH_HRM^GD03^	N	NA	NA
10	GD57	IS	Serum	BPsΔ*33*HTH_HRM^GD03^	Y (7 d)	NA	NA
				Itos	Y (7 d)	NA	NA
11	GD68	IC	Serum	Muddy	Y (1 m)	3.7	4.3 (3 m)
12	GD82	IC	Serum	Muddy	Y (2 m)	2.9	4.9 (5 m IV)
				BPsΔ*33*HTH_HRM10	Y (2 m)	0	5.3 (5 m neb)
				ZoeJΔ*45*	Y (2 m)	1.7	4.7 (5 m neb)
			Sputum	Muddy	N	NA	2.9 (3 m neb)
				BPsΔ*33*HTH_HRM10	N	NA	2.9 (3 m neb)
				ZoeJΔ*45*	N	NA	2.9 (3 m neb)
13	GD102	IC	Serum	Muddy	N	NA	NA
14	GD113	IC	Serum	BPsΔ*33*HTH_HRM^GD03^	W (7 d)	NA	NA
					W (14 d)	NA	NA
				D29_HRM^GD40^		NA	NA
15	GD116	IC	Serum	BPsΔ*33*HTH_HRM10	Y (149 d)	3.3	4.0 (269 d)
				D29_HRM^GD40^	W (269 d)	3.2	3.7 (149 d)
16	GD153	IS	Serum	Muddy	Y (17 d)	2.3	4.4 (16 wk)
17	GD156	IC	Serum	Muddy	N	NA	NA
18	GD158	IC	Serum	Muddy	Y (45 d)	2.7	5.0 (15 w IV)
19	GD194	IC	Serum	BPsΔ*33*HTH_HRM10	Y (8 wk)	NA	NA
				Itos	Y (8 wk)	NA	NA
20	BCG	MSMD	Serum	Muddy	N	NA	NA
				D29	N	NA	NA
				FionnbharthΔ*43*Δ*45*	N	NA	NA
				Fred313cpmΔ*33*	N	NA	NA

Abbreviations: BAL, bronchoalveolar lavage fluid; BCG, bacille Calmette-Guerin; ELISA, enzyme-linked immunosorbent assay; IC, immunocompetent; IS, immunosuppressed; IV, intravenous; MSMD, Mendelian susceptibility to mycobacterial disease; N, no; NA, not applicable; neb, nebulization; W, weak; Y, yes.

Only data for patients for whom serum or sputum was available.

Binary indicator of neutralization. Neutralization is defined as >10^3^ reduction in titer in 24 hours. Time after phage initiation of the earliest sample available with neutralization is shown in parentheses. No pre-phage samples were neutralizing for any patient.

Serum ELISAs show immunoglobulin G responses; sputum ELISAs show immunoglobulin A responses. Maximum observed ELISA titer at time after phage initiation is shown in parentheses.

## RESULTS

### Personalization of Bacteriophage Regimens

**Figure 1.**
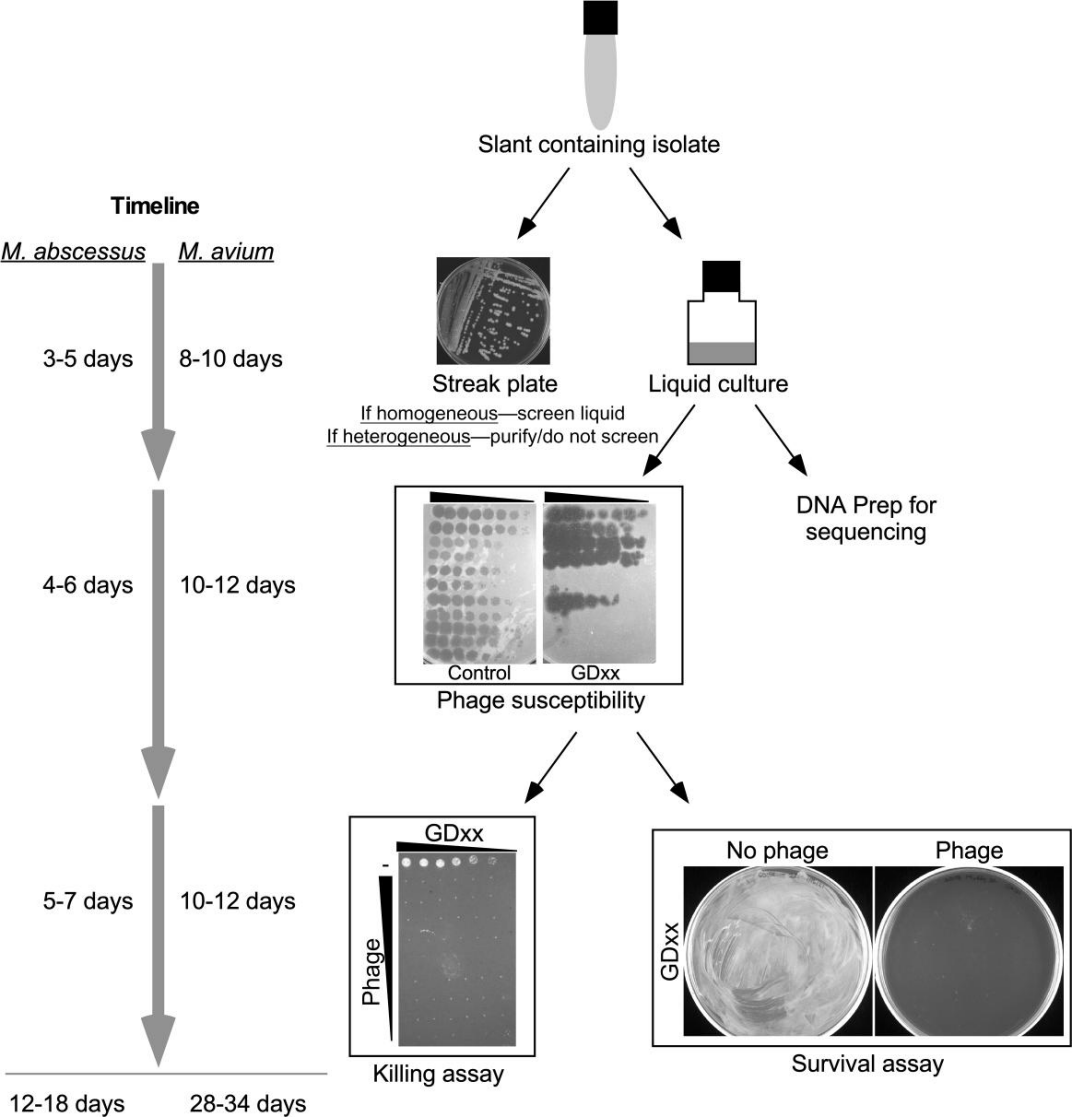
Scheme for identifying therapeutically suitable mycobacteriophages. Clinical isolates on slants (top) are cultured in liquid and streaked on solid media to determine colony morphotype and homogeneity. If both smooth and rough colony morphotypes were observed, these are colony purified and subsequently cultured and tested. If the slant appeared homogeneous, the liquid culture was used to screen again a panel of phages, determining the efficiency of plaquing (EOP) on the bacterial isolate relative to *Mycobacterium smegmatis* (control); each strain was given a GDxx identifier. Phages infecting with an EOP >0.1 were then tested in a killing assay over a range of bacterial phage concentrations and in a survival assay indicating the efficiency of killing and the likelihood of phage resistance emerging. The approximate timeline of screening is shown at left.

Mycobacterial isolates from individual patients were tested for sensitivity to a panel of approximately 25 phages representing genomic clusters known to infect *M. abscessus* or *Mycobacterium tuberculosis* (Figure 1) [5, 17, 21]. Of the 200 strains screened between May 2019 and May 2021, 157 (78%) were *M. abscessus*; 77 and 71, respectively, had rough and smooth colony morphologies; 9 were mixed and both types were purified and cultured. Fifty-five (71%) rough colony strains were infected and killed efficiently by at least 1 phage. If an isolate was efficiently infected and killed by 1 or more phage, and the patient’s clinical status indicated eligibility for compassionate use intervention as previously described, regulatory permissions were obtained and purified phages were dispatched to a local dispensing pharmacy. Of the 20 patients offered treatment, 17 had *M. abscessus* infections; 14 of these had underlying CF, 1 had bronchiectasis, 1 had scleroderma, and 1 had hypersensitivity pneumonitis. One patient had a *Mycobacterium chelonae* disseminated skin infection, 1 had CF with pulmonary *Mycobacterium avium*, and 1 had a disseminated BCG infection (Table 1). Of the 17 *M. abscessus* strains isolated, 12 were subspecies *abscessus* and 5 were subspecies *massiliense* (Table 1).

### Patient Outcomes

All patients were treated based on compassionate use, had disparate underlying conditions, and had complex infections due to diverse mycobacterial species with differing patterns of phage and antimicrobial susceptibility. Therefore, fixed definitions of treatment response were not possible. Generally, a favorable response was defined as mycobacterial smear and culture conversion to negative in at least 1 relevant specimen coupled with clinical and/or radiographic improvement or resolution of signs and symptoms of infection after at least 6–8 weeks of phage treatment. A partial response was defined as either mycobacterial smear or culture conversion or clinical and radiographic improvement. All patients had a previous history of prolonged or relapsing mycobacterial infections often coupled with other drug-resistant bacterial coinfections, underlying organ system consequences of CF or chronic lung disease, and numerous other clinical complications. Patients with CF were generally not receiving or had failed CF transmembrane conductance regulator modulators. Antibiotic therapies were optimized where possible, although most patients had only 1 or 2 at least partially active antibiotics that were used with phages. Underlying conditions and drug toxicities complicated interpretation of phage efficacy in several patients. Within this context, of the 20 patients treated, favorable or partially favorable responses were observed for 11 patients, 5 had inconclusive outcomes, and 4 had no response (Tables 1–3). Groups of patients with differing responses to phage treatment are described below; detailed synopses are in the Supplementary Information.

#### Favorable Clinical or Microbiological Responses

Favorable clinical and microbiological responses were observed in 5 patients (patients 1, 9, 10, 15, and 16) and partial clinical or microbiological responses were noted in 6 patients (patients 2, 4, 7, 13, 17, and 20) (Table 1); all were treated with IV phages except for patients 13 and 17, who also received nebulized phages after the IV regimen. Eight of these 11 patients had CF and complex pulmonary infections (7 *M. abscessus*, 1 *M. avium*), 1 had disseminated skin lesions caused by *M. chelonae*, 1 had scleroderma with *M. abscessus* infection, and 1 had disseminated BCG infection (Table 1). In 5 patients (patients 9, 10, 15, 16, and 17), the infections appear to have been largely resolved: 3 with pulmonary *M. abscessus* infections (patients 9, 10, and 15), 1 with pulmonary *M. avium* infection (patient 17), and another with *M. chelonae* skin infection (patient 16); details of patients 15 and 16 were recently reported [22, 23]. Three of these 11 patients (patients 7, 16, and 17) had no change in their antibiotic regimen during phage treatment, but at least 4 had some change to their antibiotic course. Patient 15 cleared *M. abscessus* cultures and successfully underwent a bilateral lung transplant. Two patients (1 and 13) had substantial clinical improvement, but without clear evidence of culture conversion. One of these (patient 1) was the case reported previously [5] in which posttransplant disseminated *M. abscessus* infection and clinical signs and symptoms greatly improved, but some skin nodules persisted after >1 year of phage treatment. These were only intermittently culture positive, but the patient subsequently died from CF-related health challenges and organ failure 44 months after the start of phage treatment. In the other (patient 13), there was substantial improvement of symptoms and forced expiratory volume in 1 second by spirometry, but the patient remained culture positive.

For 4 patients (patients 2, 4, 7, and 20), response to therapy was partial and more difficult to assess largely due to complications from other infections, although there was evidence of improved control of the *Mycobacterium* infections. One patient (patient 2) had a severe chest infection requiring sternum resection. Phage treatment resulted in AFB smear-negative chest swabs, but the patient died after failing therapy for multiple bacterial and fungal coinfections. For patient 4, phage treatment resulted in conversion to culture negative tracheal aspirates, but systemic adenovirus infection resulted in death. Patient 7 had *M. abscessus* infection with both rough and smooth colony morphotypes; the rough strain derived from the smooth strain by a mutation in glycopeptidolipid synthesis [17]. The phage identified for the rough strain (Table 1) did not infect the smooth counterpart but was administered on the possibility that removal of the rough variant could be clinically beneficial, even if the smooth strain persisted. The patient remained clinically stable but had persistently positive sputum cultures that grew only smooth strains after phage administration, suggesting that phage treatment had reduced the burden of the rough strain. Finally, patient 20 had MSMD and disseminated BCG infection. Phage treatment resulted in improved clinical signs and symptoms and marked reduction in BCG PCR positivity in weekly blood and urine samples. However, the patient died of other complications.

#### Inconclusive or Incomplete Outcomes

Five patients (patients 3, 8, 11, 12, and 18) had inconclusive responses to therapy or had modest short-lived improvements. Patient 8 developed phage neutralizing antibodies and had little clinical improvement with either aerosolized or IV administered phage, likely due to the phage neutralizing immune response. Like patient 7, patient 11 had a mixed infection with both rough and smooth colony morphotypes of *M. abscessus*, and active phage was identified only for the rough strain (Table 1). While chest radiographs improved, sputum cultures were intermittently positive. Patient 12 showed some reduction in sputum *M. abscessus* load during the first month of IV administration, but subsequent recrudescence correlated temporally with an increase in antibody-mediated phage neutralization [19]. Patient 18 has not shown substantial clinical improvement, likely also due to serum neutralization of phage; he has been switched to aerosolized phage therapy after 4 months of IV administration and treatment is ongoing. Patient 3 died shortly after the start of phage administration due to multiple organ failure.

#### No Clinical or Microbiological Improvement

Three patients with CF and 1 with non-CF bronchiectasis (patients 5, 6, 14, and 19), all with pulmonary *M. abscessus* infections, showed no overall clinical or microbiologic response (Table 1). All had been treated with IV phage, although 1 patient (patient 19) was switched to aerosolized administration. The reasons for lack of response are unclear.

### Safety, Resistance, and Immunity

Phage administration by either IV or aerosolized routes was well-tolerated with no serious adverse reactions related to the phage in any patient. Phage preparations were highly purified, certified to be sterile, and had undetectable endotoxin levels, an advantage of using a lipopolysaccharide-free bacterial host (*M. smegmatis*) for phage growth. Eleven patients were treated with just a single phage (Table 1; 8 with Muddy, 3 with BPsΔ*33*HTH_HRM^GD03^), and in the 6 patients from whom rough colony *M. abscessus* isolates were recovered after the start of phage treatment, all remained fully phage sensitive. Indeed, changes in phage sensitivity were only observed in 1 phage in a cocktail of 3 (Table 2, patients 1 and 12).

Sera from 15 patients were tested for immune reactions, and robust neutralization of at least 1 phage was observed in 8 of the patients following IV treatment (Table 3). Although neutralization correlated temporally with loss of clinical response in patient 12 [19], there was no correlation between neutralization and outcomes in 8 remaining patients. Patients 8, 18, and 19 also developed neutralizing antibodies, plausibly contributing to reduction in therapeutic response, but strong neutralization to at least 1 of the phages in patients 15 and 16 did not prevent favorable or partial responses (Tables 1 and 3). Patient 1 had no phage neutralization until 2 years after starting therapy, when only 1 of the 3 phages had decreased activity in serum. Curiously, in some patients (patients 12, 15, and 16), pre-phage sera recognized the phages, although these were not neutralizing. Presumably, this reflects prior exposure to related but distinct phages in the environment.

## DISCUSSION

This series of 20 patients treated with phages on a compassionate use basis provides support for further evaluation of phages for treatment of mycobacterial infections. Phage administration was well-tolerated, and phage resistance was not observed even when using a single phage. Favorable responses were observed in more than half of the patients, including complete resolution of some infections, and successful lung transplantation in 1 patient. However, some patients saw little clinical benefit, and the basis for this variability in response is unclear. Although phage treatment of mycobacterial infections shows promise, this cohort illustrates some key limitations and lessons.

First, the repertoire of therapeutically useful phages is small, and expansion requires further phage isolation, developing phages induced from lysogenic strains or using synthetic phages [17, 24]. However, the lack of phage resistance (Table 2) supports use of a single phage, and where >1 phage is available, cycling their administration to circumvent neutralization. Second, optimal routes of phage administration and adequacy of tissue penetration are unclear. IV administration may be preferable for treatment of disseminated infections and appears effective for at least some lung infections, particularly when there is structural lung damage due to fibrosis, severe bronchiectasis, or mucoid plugging that compromises delivery by nebulization alone. We note that for patient 9, in whom the pulmonary *M. abscessus* infection was fully resolved, the phages were also deposited bronchoscopically, which may have contributed to more effective phage delivery to the infection. Nebulization may also avoid systemic neutralization. The immune status of the patient is also important; immunocompromised patients may tolerate extended phage administration without antibody-mediated neutralization. However, little is known about intracellular penetration or uptake of phages, particularly by macrophages, where most replicating mycobacteria are found. Third, dosage and regimens warrant optimization. As the treatments are well-tolerated, higher doses could be contemplated, using longer interdose intervals. Further exploration of pharmacodynamics and tissue penetration of phage is critical.

The lack of therapeutically useful phages for smooth *M. abscessus* strains, the unpredictable specificity for rough strains, and the limited phage repertoire represent current impediments to broad implementation of phage treatments. However, these limitations are not insurmountable, and these case studies suggest that phage treatments may be valuable tools for clinical control of NTM infections. Successful outcomes for *M. chelonae*, *M. avium*, and BCGosis, as well as *M. abscessus*, suggest a large spectrum of target *Mycobacterium* diseases.

Although compassionate use case studies such as these lack the rigor and consistency of treatment and patient monitoring possible in carefully controlled blinded clinical trials, they provide a wealth of insights for designing such trials. Variations in antibiotic regimens, surgical interventions, and management of coinfections can all potentially influence patient status, and direct linkage of phage treatments with outcomes in individual patients is perilous. Nonetheless, this series of case studies strengthens the likelihood of direct benefits from phage treatments of *Mycobacterium* infections and the potential for infection control when none other is effective.

## Supplementary Material

ciac453_Supplementary_Data

## Supplementary Data

Supplementary materials are available at *Clinical Infectious Diseases* online. Consisting of data provided by the authors to benefit the reader, the posted materials are not copyedited and are the sole responsibility of the authors, so questions or comments should be addressed to the corresponding author.

## Notes

***Acknowledgments*.** The authors are very grateful to our colleagues and collaborators including Owen Abad, Haley Aull, Lindsey Baden, Megan Clancy, Jonathan D’Cunha, Aleksandra Petrovic Fabijan, Katrina Ford, Melanie Forster, Alessia Franceschi, Will Garner, Kailey Hughes, Saija Kiljunen, Jon Koff, Ken Olivier, Eero Lauhkonen, Outi Lyytinen, Diana Pakstis, Crystal Petrone, Jenni Rosendahl, Aleksandra Theeba Thiruchelvam, and Hanna Vahtera. They also thank all of the faculty and student phage hunters in the SEA-PHAGES program who contributed to the phage collection used to identify therapeutically suitable phages.

***Financial support.*** This work was supported by the National Institutes of Health (NIH) (grant number GM131729), the Howard Hughes Medical Institute (grant number GT12053), and the Cystic Fibrosis Foundation (grant number HATFUL19GO). This work was also supported by a kind donation from the Fowler Fund for Phage Research to G. F. H; the National Heart, Lung, and Blood Institute (grant number K08 HL139994-01) and the Burroughs Wellcome Fund Career Award for Medical Scientists to K. A. C.; the NIH (HL146228) and the Cystic Fibrosis Foundation (grant numbers NICK20Y2-SVC and NICK20Y2-OUT) to J. A. N.; the NIH (grant number AI154546) to G. H; the Jane and Aatos Erkko Foundation to M. S.; the Mallory Smith Legacy Fund and the University of California, San Diego Chancellor’s Fund to C. A. B. and R. T. S.; and the NIH (grant number R01HL146228) and Cystic Fibrosis Foundation (grant numbers NICK20Y2-SVC and NICK20Y2-OUT) to R. M. Da. (co-investigator).
