# Supplementary material for: Phage Therapy of Mycobacterium Infections: Compassionate Use of Phages in 20 Patients With Drug-Resistant Mycobacterial Disease
Source: Clin Infect Dis. 2022 Jun 9;76(1):103–12. doi: 10.1093/cid/ciac453 (PMC9825826; doi:10.1093/cid/ciac453)
Supplement: ciac453_Supplementary_Data [file ciac453_supplementary_data.zip › Supplementary Information Revised clean 5-23-22.docx]

**SUPPLEMENTARY INFORMATION**

**Case details**

***Patients 1 (GD01) and 10 (GD57).*** Patients 1 and 10 both have CF, underwent bilateral lung transplantation followed by initiation of immunosuppressive drugs to control rejection, and had disseminated *M. abscessus* infections. Patient 1 was described in detail previously ^5^ and had an *M. abscessus* susp. *massilense* infection that was treated with a three-phage cocktail together with her current antibiotic regimen. Three years after treatment initiation, the patient died due to multiple transplant complications. Patient 10 has CF and an *M. abscessus* infection established after lung transplantation. They were treated with a two-phage cocktail (Table 3) in addition to their ongoing antibiotic regimen. After one month of treatment, spirometry values improved, the patient gained weight, had no further hospitalizations, and liver function was improved. PET/CT scans after five months and then one year of treatment showed no disseminated infection or new infection locations. Occasional sputum samples were culture positive, but phage administration was stopped after one year, as chest scans were clear, and the patient had sustained improvement in lung function as well as a negative bronchoalveolar culture that was negative after one year.

***Patient 2 (GD10)*.** Patient 2 had scleroderma and underwent bilateral lung transplantation and developed an *M. abscessus* infection post-operatively, which disseminated to the chest and skin and was antibiotic refractory. The sternum was removed due to severe *M. abscessus* infection. Phage Muddy was administered intravenously and used for chest irrigation (100mls) in the operating room and topical application to the skin nodules, with a total dose of 10^8^ pfu. Muddy was also administered intravenously at a dose of 10^9^ pfu by IV twice daily. *M. abscessus* was recovered from chest swabs at days 3, 10 and 13 after phage administration. These were grown, tested, and shown to still be susceptible to Muddy. PCR was used to check serum and for phage, but none were positive. The patient received 4 weeks of phage therapy. Although most clinical cultures remained positive, their AFB smear burden decreased: all samples were heavily smear-positive prior to phage administration and became either smear-negative or ‘rarely positive’ after phage. Unfortunately, the patient declined shortly thereafter with sepsis and a disseminated fungal infection. The family withdrew care, and the patient passed away.

***Patient 3 (GD20).*** Patient 3 is an adult with CF and a chronic pulmonary *M. abscessus* disease. A two-phage cocktail was administered intravenously but stopped after two days due to a generally worsening condition. Phage administration was restarted several days later, but the patient died due to multiple organ failure after another seven days.

***Patient 4 (GD24).*** Patient 4 has CF and had bilateral lung transplantation, and refractory pulmonary *M. abscessus* disease. A single phage was administered IV, and approximately one month after starting phage treatment, tracheal aspirates were *M. abscessus* culture negative. However, the patient died soon after due to a systemic adenovirus infection with severe acute respiratory distress syndrome.

***Patients 5 (GD25) and 6 (GD40)*.** Both patients have CF with severe antibiotic-refractory pulmonary *M. abscessus* disease. A single phage was administered IV for each patient for at least three months. Patient 5 remained smear positive after three months and chose to stop treatment, but subsequently decided to restart phage IV administration, saw little or no clinical improvement, and five months later switched to nebulized phage treatment. No clinical improvement was observed after another five months of treatment. Patient 6 similarly saw no consistent clinical improvement after six months of phage administration and treatment was stopped.

***Patients 7 (GD43) and 11 (GD68).*** Patients 7 and 11 are both pediatric patients with CF and pulmonary *M. abscessus* disease (Table 1). For both patients, both rough and smooth colony morphotype strains were isolated from respiratory secretions, which were shown to differ from each other only by mutations in *mps1*^19^. Neither of the smooth strains were efficiently killed by any phages tested, but the rough strains were killed by a single phage. Therapy was initiated with this phage given IV twice daily at 10^9^ PFU/dose. Patient 7 remained clinically stable but sputum continued to isolate *M. abscessus*, although post-treatment isolates showed only smooth colony types, suggesting that phage treatment had at least reduced the burden of infection of the rough morphotype. Patient 11 showed some clinical improvement over 18 months of therapy, with improved chest CT scans, but remained AFB smear positive, although with intermittently positive cultures. Both rough and smooth *M. abscessus* morphotypes were recovered post-treatment, and the rough isolate remained sensitive to Muddy. Lung function did not improve substantially, and AFB smears of respiratory secretions remained positive. Serum collected during treatment showed neutralization of Muddy *in vitro* with maximal inactivation after 6-7 months of treatment but some reduction in neutralization after another seven months. Nebulized administration of phage is being considered.

***Patient 8 (GD45).*** A CF patient with antibiotic-refractory *M. abscessus* pulmonary disease was treated with a single phage, Muddy, administered initially by nebulization. After two months of treatment, the chest CT scan was unchanged and the patient remained AFB smear and culture positive, although they self-reported a sense of improvement. The nebulized dose was increased to 10^10^ PFU/dose and IV administration was added. No substantial clinical improvement was observed. Curiously, a 6-month post-treatment *M. abscessus* isolate remained sensitive to Muddy but had newly acquired sensitivity to phage BPs derivatives. Sequencing of the pre- and 6-month post-treatment *M. abscessus* isolates showed a substantial number of single nucleotide polymorphisms (>120) and a deletion, as well as loss of plasmid pGD45-1 (Table S1). Serum samples 4-6 months post IV/nebulized therapy showed complete neutralization of Muddy *in vitro*. The patient has recently started aerosol administration of Muddy and BPs∆*33*HTH_HRM10.

***Patient 9 (GD54).*** Patient 9 has CF with antibiotic refractory *M. abscessus* pulmonary disease resulting in a ~40% decline in lung function as measured by spirometry FEV_1_. The patient previously had high dose inhaled nitric oxide treatment in addition to two years of aggressive antibiotic therapy but remained intermittently AFB smear positive, and persistently culture-positive from sputum and bronchiolar lavage (BAL) samples. A two-phage cocktail was administrated intravenously in addition to a new regimen of antibiotics, and phage was also delivered bronchoscopically twice at the start of treatment. Multiple sputum samples collected after the start of therapy were all culture negative for *M. abscessus* as well as BAL samples collected 3 weeks and 10 months into the course of therapy. Phage therapy was stopped after 10-months of administration, and antibiotic treatment was stopped two months after that. No *M. abscessus* has since been detected including on BAL culture collected 3 months after ceasing all antimicrobials and they are considered free of the infection.

***Patient 12 (GD82).*** Patient 12 has non-CF bronchiectasis and phage treatment was previously reported^18^. In brief, a 1.2 log reduction in *M. abscessus* sputum counts was seen after 1 month of phage administration, but the bacteria rebounded to over pre-therapy levels due to antibody-mediated phage neutralization. Post treatment *M. abscessus* isolates remained fully sensitive to phage Muddy and BPs∆*33*HTH_HRM10, but partial resistance to phage ZoeJ∆*45* was observed in some isolates^18^. After six months, treatment was switched to aerosolized delivery, which continued for nearly 12 months. Due to ongoing overall health decline, the patient was transitioned to hospice, yet continued aerosol phage administration through the terminal illness. Ultimately, the patient deceased in home hospice.

***Patient 13 (GD102).*** This patient has CF and an antibiotic refractory *M. abscessus* pulmonary infection; single phage therapy was administered intravenously. After three months of phage treatment the patient remained AFB smear positive, but with generally improved clinical signs. After 10 months of phage treatment, *M. abscessus* isolates remain phage-sensitive and neutralization is not detected. The patient was switched to aerosolized phage for four weeks and phage treatment is now on hold.

***Patient 14 (GD113).*** This patient has hypersensitivity pneumonitis and *M. abscessus* lung disease. The patient had stents and multiple dilations previously, but also had bilateral bronchial stenosis. The *M. abscessus* isolate was found to be susceptible to derivatives of phage BPs and D29. The patient was treated by IV at 10^9^ PFU/dose of the BPs derivative and 10^8^ PFU/dose of the D29 derivative, twice daily. After starting on phage treatment, the patient required fewer bronchoscopies and bronchial dilatations but remained NTM culture positive.

***Patient 15 (GD116).*** Patient 15 has bronchiectasis secondary to CF and an antibiotic refractory *M. abscessus* pulmonary disease. A two-phage cocktail was administered intravenously and although cultures from respiratory secretions remained positive through day-95 of phage treatment, subsequent cultures converted to negative. After 12 months of phage administration, the patient was accepted for and underwent lung transplantation. Systematic culturing of the explanted lung did not demonstrate viable *M. abscessus*. The patient has continued on phage and antibiotic treatment post-transplant without evidence of recurrent infection. Details of this case have been submitted for publication.

***Patient 16 (GD153).*** Patient 16 has an autoimmune disorder with a disseminated *M. chelonae* skin infection presenting as skin lesions and subcutaneous nodules on several limbs. Phage Muddy was administered intravenously in addition to an optimized antimicrobial regimen and after three months of treatment, the skin lesions were largely resolved, one biopsy was negative for *M. chelonae* and showed no evidence of granuloma, and a PET/CT scan showed significant improvement. An additional biopsy was negative for AFB and treatment is concluding.

***Patient 17 (GD156).*** Patient 17 has CF and pulmonary *M. avium* disease. The bacterial isolate is sensitive to phage Muddy, but when the strain was challenged with phage *in vitro*, killing was less complete than typically observed, with many surviving mycobacteria. Notwithstanding concerns about the potential risk for phage resistance *in vivo*, phage was administered IV due to poor clinical status. After twelve days of phage treatment, the patient’s FEV_1_ increased to 61%, although it had not been above 47% for the prior two years. Serum from 12 days post-therapy was found to have modest neutralization activity (Table 4). The patient was switched to aerosol delivery of the phage after 3 months. All AFB smears have been negative and AFB culture time to positivity increased from 1 week prior to treatment to 4 - 8 weeks after treatment. While the morphology looks similar to the previously isolated MAC, confirmation is still pending at this time. Consecuative AFB cultures obtained at 21 and 25 weeks after treatment have shown no growth.

***Patient 18 (GD158).*** Patient 18 has CF and antibiotic refractory *M. abscessus* pulmonary disease. Phage Muddy was administered intravenously. After 6-7 weeks of phage, the patient contracted respiratory syncytial virus. Two post-treatment *M. abscessus* isolates were still sensitive to the phage. Serum samples obtained after nine weeks of therapy showed near complete neutralization. After 4 months of IV treatment, aerosolized phage was added to IV administration (Table 4).

***Patient 19 (GD194).*** Patient 19 has CF and antibiotic refractory *M. abscessus* lung disease. Phages were administered intravenously, and the patient showed no substantial clinical improvement, and a strongly neutralizing antibody response was observed eight weeks after initiation of treatment. Phage delivery has recently been switched to aerosol administration.

***Patient 20*.** Patient 20 has a heterozygous mutation in *NFKBIA* gene c.32 G>A; p.(Trp11*) Mendelian Susceptibility to Mycobacterial Diseases (MSMD) and had been vaccinated with BCG at birth but developed disseminated BCG infection with associated hyperinflammation. The BCG strain was not successfully cultured at this time, although BCG was consistently detected by PCR in both blood and urine at high cycle threshold. Phage susceptibilities differ little among BCG strains (our unpublished observations) and phages likely to be active were delivered by IV. Clinical improvement, with significant reduction in fever and inflammatory markers was observed for approximately 6 months. BCG PCR testing in both blood and urine became negative 1 month after initiation of phage treatment. Unfortunately, 6 months after treatment initiation and following the cessation of anti-mycobacterial antibiotics, the patient developed BCG pneumonia and peritonitis (BCG culture and PCR positive). Although these infections were subsequently controlled with combined phage therapy and anti-mycobacterial antibiotics, the patient subsequently died of other complications.

**Supplementary Methods**

**Phage susceptibility determination and preparation for clinical use**

Upon receipt of clinical isolates of mycobacteria, homogeneity was assessed by growth on solid medium (Middlebrook 7H10) at 37° C, and single colonies were used to inoculate 10 mls Middlebrook 7H9 media with OADC (Oleic Albumin Dextrose Catalase) and 1 mM CaCl_2_ and incubated with shaking at 37°C until grown to an optical density measured at 600 nm of between 0.5 and 1, typically 3-5 days for *M. abscessus* or 8-10 days for *M. avium*. Cultures were dispersed by brief sonication (Q-sonica 500 at 30% amplitude with 15 sec on and 10 sec off until visibly dispersed) as described previously ^5^. Plaque assays were used to determine phage susceptibility profiles, spotting 3 µl 10-fold serial dilutions on top of agar bacterial lawns. Efficiencies of plaquing (EOP) were determining by comparing phage titers on a clinical isolate with infection of *M. smegmatis* mc^2^155, as described previously ^20^.

Phages with an EOP >0.1 were further evaluated for the ability to kill the clinical isolates over a range of bacterial and phage concentration, by incubating in liquid culture followed by plating on solid media to determine bacterial survival, as described previously ^5,19^. If no bacterial growth was observed over a broad range of conditions, we also tested if the phage efficiently kills ~5 x 10^7^ Colony forming units (CFU) at an MOI of ten by incubating in liquid culture and plating for growth on solid media.

Phage preparations for clinical use were amplified on *M. smegmatis* mc^2^155, concentrated by centrifugation, banded twice by CsCl equilibrium density centrifugation, and extensively dialysized, as described previously ^5,18^. Phages were distributed to a total of 0.1 ml total with 10^11^ PFU/ml of each phage into sterile sealed vials and analyzed for endotoxin activity by EndoZyme II assay (Hyglos GmbH), and for sterility certification by Accugen Inc. Phages were stored at 4°C and periodically tested for stability.

**ELISA and neutralization assays**

Enzyme-linked immunosorbant (ELISAs) and phage neutralization assays were performed as described previously (18).

**PCR analysis**

PCR analysis to detect phage DNA was performed using either 1 µl total DNA (250 ng) extracted from sputum using DNA Isolation Kit (Norgen Inc) or 1 µl serum, and phage-specific DNA oligonucleotide primers; all reactions used Q5 High Fidelity 2X Master Mix (New England Biolabs Inc.). Primers for phage BPs yielded either a 2.3 Kbp product: 5’-TGGAAGAACGCGCGCTGCTC-3’ and 5’-GCGCGAACCCGACGGCAATC-3’) or a 566 bp product (5’-GGCCGTGGGCAGAGGAAACC-3’

and 5’-AGAACCTCAACACCGGCGCG-3’); for phage Itos, primers amplified a 577 bp product (5’-tggtaggccactccctcaat-3’ and 5’-gtggttacctaagtccggtaggagacac-3’); for phage Muddy, primers amplified a 745 bp product (5'-CGCTGATGCTACAAGGTTTTAC-3' and 5'-GCCGTTGACATACCAGACG-3'); and for phage D29, primers amplified a 254 bp product (5’-gattctcactctaccggactagtc-3’ and 5’-CTTGCTGCGAtGACACAAGTAAAC-3’). Products were analyzed by agarose gel electrophoresis.

**Regulatory considerations**

Permission for compassionate use was obtained by the treating physicians through an expedited investigational new drug (eIND) application to the FDA and local IRB approval within the US, and related processes in other countries.

**Table S1. Minimum inhibitory concentrations of NTM clinical isolates.**

| **Patient #** | **Strain** | **AMI** | **FOX** | **CIP** | **CLA** | **DOX** | **IMI** | **LZD** | **MC** | **MXF** | **TGC** | **TMP-SMX** | **TOB** | **BDQ** | **CFZ** |
| --- | --- | --- | --- | --- | --- | --- | --- | --- | --- | --- | --- | --- | --- | --- | --- |
| 1 | GD01 | >64 | 128 | >8 | >16 | >16 | ND | 32 | ND | >8 | ND | ND | ND | ND | ND |
| 2 | GD10 | S | I | R | S | R | I | S | R | R | 0.25 | R | ND | ND | ND |
| 3 | GD20 | R | >128-R | R | R | R | 16-I | R | ND | R | 2-S | S | R | ND | <0.5-S |
| 4 | GD24 | ND | ND | ND | ND | ND | ND | ND | ND | ND | ND | ND | ND | ND | ND |
| 5 | GD25 | >64-R | 4-R | 4-R | >16-R | >16-R | 8-I | 4-S | >8-R | 4-R | 0.25 | 8/152-R | >16 | ND | ND |
| 6 | GD40 | 4-S | 64-I | >4-R | >16-R | >16-R | 16-I | 4-S | ND | 8-R | 0.25 | >8/152-R | ND | ND | ND |
| 7 | GD43 | 4-S | 32-I | 2-I | 2-S | 2-I | >64-R | 8-S | >8 | 2-I | 0.25 | ND | ND | ND | ND |
| 8 | GD45 | >64-R | 64-I | >8-R | >32-R | >16-R | >16-TR | 16-I | >8-TR | >4-R | 2-S | >4/78-R | 16-R | ND | ≤0.5-TS |
| 9 | GD54 | S | I | R | R | R | R | S | ND | R | ND | R | ND | ND | ND |
| 10 | GD57 | >64-R | >128-R | 4-R | >16-R | >16-R | NT | 8-S | >8-R | 8-R | ND | >8/152-R | ND | ND | ND |
| 11 | GD68 | 16-S | 32-I | 4-R | >16-R | 2-I | 16-I | <1-S | <=1-S | 0.5-S | ND | ND | ND | ND | ND |
| 12 | GD82 | 32 | 64-I | >4-R | >16-R | >16-R | 64-R | 32-R | >8-R | >8-R | 4 | >8/152-R | >16-R | ND | ND |
| 13 | GD102 | >64-R | 32-I | 4-R | >16-R | >16-R | 8-I | 4-S | >8 | 4-R | 0.25 | 2-S | ND | ND | ND |
| 14 | GD113 | >64-R | 64-I | >4-R | >16-R | >16-R | ND | 16-I | >8-R | 8-R | ND | >8/152-R | ND | ND | ND |
| 15 | GD116 | 16-S | 32-I | >8-R | 1-S | 16-R | 16-I | 16-I | >8-R | >4-R | 1 | >4/76-R | 8 | ND | ≤0.5-TS |
| 16 | GD153 | 16-S | 128-R | >4-R | >16-R | >16-R | >64-R | 4-S | >8-R | 4-R | 0.12 | 1/19-S | ≤1-S | 0.001 | <0.015 |
| 17 | GD156 | 8-S | ND | >8-NI | 4-S | >8-NI | ND | 32-R | >8-NI | >4-R | ND | 2/38-NI | ND | ND | 0.12-NI |
| 18 | GD158 | ≤8-S | 64-I | >8-R | >32-R | >16-R | >16-R | >16-R | >8-R | >4-R | 2-S | >4/78-R | 8-R | ND | ≤0.5-S |
| 19 | GD194 | >256-R | >128-R | >4-R | >16-R | >8-R | >32-R | 32-R | ND | >4-R | 0.5 | 4/76-R | ND | ND | ND |
| 20 | BCG | ND | ND | ND | ND | ND | ND | ND | ND | ND | ND | ND | ND | ND | ND |

Minimum inhibitory concentrations (MIC) are shown for each clinical isolate determined at or prior to the start of phage therapy; values are in µg/ml. Interpretations of the strain phenotype are also shown including when MICs are not available, as follows: S=sensitive, I=intermediate, R=resistant, TR=tentatively resistant, TS=tentatively sensitive, ND=not determined, NI=not interpretable. Antibiotics are as follows: Amikacin (AMI), Cefoxitin (FOX), Ciprofloxacin (CIP), Clarithromycin (CLA), Doxycycline (DOX), Imipenem (IMI), Linnezolid (LZD), Minocycline (MC), Moxifloxacin (MXF), Tigecycline (TGC), Trimethoprim/Sulfamethoxazole (TMP-SMX), Tobramycin (TOB), Bedaquiline (BDQ), Clofazimine (CFZ).

**Supplementary Figure Legends**

**Figure S1. PCR detection of phage DNA in clinical samples.** PCR analysis of phage DNA in serum or sputum samples from eight patients (panels A-H). Each panel (A-H) shows a DNA marker ladder (L), a positive control (+) using phage lysate as input DNA, and negative control (-) with no input DNA. **A**. Patient #3 samples amplified with BPs or Itos primers. Lanes 1-4 contain serum 1, 6, 12, 24 hr post phage infusion; Lane 5 - pre-treatment serum; Lanes 6-10 - sputum DNA 30 min, 2, 5, 12 and 24 hr post phage infusion. **B**. Patient #4 samples amplified with BPs primers. Lanes 1-2 - serum 5 and 7 days after first phage infusion; Lanes 3-4 - tracheal aspirates during phage treatment; Lane 5 - BAL sample during phage treatment. **C**. Patient #5 samples amplified with Muddy primers. Lanes 1-6 -serum samples pre-phage, 30 min, 1, 2, 4 and 6 hr after phage infusion. **D**. Patient #8 samples amplified with Muddy primers. Lanes 1-4 - DNA from sputum samples days 2, 14, 28, 35; Lane 5 - serum after 35 days of phage treatment. A weakly positive sample is seen in Lane 4 (Day 35 sputum). **E**. Patient #9 samples amplified with Muddy and BPs primers. Lane 1 - pre-phage serum; Lanes 2-7 - serum 2, 4, 8, 11, 15, and 25 days after the start of phage treatment, Lanes 8-10 - serum from 32 days after the start of phage treatment at 20, 1 and 2 hr after phage infusion; Lane 11 - serum 39 days after phage treatment, Lanes 12-14 - serum 43 days after phage treatment at 20, 1 and 2 hr after phage infusion. Two positive samples are seen in Lanes 13 and 14, corresponding to 1 and 2 hours after phage infusion on day 43. **F**. Patient #11 samples amplified with Muddy primers. Lanes 1-9 - pre-phage serum, 15 days, 30 days, 2, 3, 8, 9, 10, 11 months after the start of phage treatment. Two positive samples are seen in Lanes 2 and 5, corresponding to 15 days and 3 months after the start of phage treatment. **G**. Patient #13 samples amplified with Muddy primers. Lanes 1-6 - pre-phage serum, 1, 7, 14, 21 and 42 days after the start of phage treatment. **H**. Patient #15 samples amplified with BPs and D29 primers. Lane 1 - pre-phage serum, Lane 2 - 5 min after phage infusion, Lanes 3-7 serum from 3, 47, 82, 126 and 152 days after the start of phage treatment.
