# Supplementary material for: Phage Therapy of Mycobacterium Infections: Compassionate Use of Phages in 20 Patients With Drug-Resistant Mycobacterial Disease
Source: Clin Infect Dis. 2022 Jun 9;76(1):103–12. doi: 10.1093/cid/ciac453 (PMC9825826; doi:10.1093/cid/ciac453)
Supplement: ciac453_Supplementary_Data [file ciac453_supplementary_data.zip › Table S1.pdf]

Table S1. Minimum inhibitory concentrations of NTM clinical isolates.

Patient #	Strain	AMI	FOX	CIP	CLA	DOX	IMI	LZD	MC	MXF	TGC	TMP-SMX	TOB	BDQ	CFZ
1	GD01	>64	128	>8	>16	>16	ND	32	ND	>8	ND	ND	ND	ND	ND
2	GD10	S	I	R	S	R	I	S	R	R	0.25	R	ND	ND	ND
3	GD20	R	>128-R	R	R	R	16-I	R	ND	R	2-S	S	R	ND	<0.5-S
4	GD24	ND	ND	ND	ND	ND	ND	ND	ND	ND	ND	ND	ND	ND	ND
5	GD25	>64-R	4-R	4-R	>16-R	>16-R	8-I	4-S	>8-R	4-R	0.25	8/152-R	>16	ND	ND
6	GD40	4-S	64-I	>4-R	>16-R	>16-R	16-I	4-S	ND	8-R	0.25	>8/152-R	ND	ND	ND
7	GD43	4-S	32-I	2-I	2-S	2-I	>64-R	8-S	>8	2-I	0.25	ND	ND	ND	ND
8	GD45	>64-R	64-I	>8-R	>32-R	>16-R	>16-TR	16-I	>8-TR	>4-R	2-S	>4/78-R	16-R	ND	≤0.5-TS
9	GD54	S	I	R	R	R	R	S	ND	R	ND	R	ND	ND	ND
10	GD57	>64-R	>128-R	4-R	>16-R	>16-R	NT	8-S	>8-R	8-R	ND	>8/152-R	ND	ND	ND
11	GD68	16-S	32-I	4-R	>16-R	2-I	16-I	<1-S	<=1-S	0.5-S	ND	ND	ND	ND	ND
12	GD82	32	64-I	>4-R	>16-R	>16-R	64-R	32-R	>8-R	>8-R	4	>8/152-R	>16-R	ND	ND
13	GD102	>64-R	32-I	4-R	>16-R	>16-R	8-I	4-S	>8	4-R	0.25	2-S	ND	ND	ND
14	GD113	>64-R	64-I	>4-R	>16-R	>16-R	ND	16-I	>8-R	8-R	ND	>8/152-R	ND	ND	ND
15	GD116	16-S	32-I	>8-R	1-S	16-R	16-I	16-I	>8-R	>4-R	1	>4/76-R	8	ND	≤0.5-TS
16	GD153	16-S	128-R	>4-R	>16-R	>16-R	>64-R	4-S	>8-R	4-R	0.12	1/19-S	≤1-S	0.001	<0.015
17	GD156	8-S	ND	>8-NI	4-S	>8-NI	ND	32-R	>8-NI	>4-R	ND	2/38-NI	ND	ND	0.12-NI
18	GD158	≤8-S	64-I	>8-R	>32-R	>16-R	>16-R	>16-R	>8-R	>4-R	2-S	>4/78-R	8-R	ND	≤0.5-S
19	GD194	>256-R	>128-R	>4-R	>16-R	>8-R	>32-R	32-R	ND	>4-R	0.5	4/76-R	ND	ND	ND
20	BCG	ND	ND	ND	ND	ND	ND	ND	ND	ND	ND	ND	ND	ND	ND

Minimum inhibitory concentrations (MIC) are shown for each clinical isolate determined at or prior to the start of phage therapy; values are in µg/ml. Interpretations of the strain phenotype are also shown including when MICs are not available, as follows: S=sensitive, I=intermediate, R=resistant, TR=tentatively resistant, TS=tentatively sensitive, ND=not determined, NI=not interpretable. Antibiotics are as follows: Amikacin (AMI), Cefoxitin (FOX), Ciprofloxacin (CIP), Clarithromycin (CLA), Doxycycline (DOX), Imipenem (IMI), Linnezolid (LZD), Minocycline (MC), Moxifloxacin (MXF), Tigecycline (TGC), Trimethoprim/Sulfamethoxazole (TMP-SMX), Tobramycin (TOB), Bedaquiline (BDQ), Clofazimine (CFZ).
