# Supplementary figures and images for: Phage Therapy of Mycobacterium Infections: Compassionate Use of Phages in 20 Patients With Drug-Resistant Mycobacterial Disease
Source: Clin Infect Dis. 2022 Jun 9;76(1):103–12. doi: 10.1093/cid/ciac453 (PMC9825826; doi:10.1093/cid/ciac453)

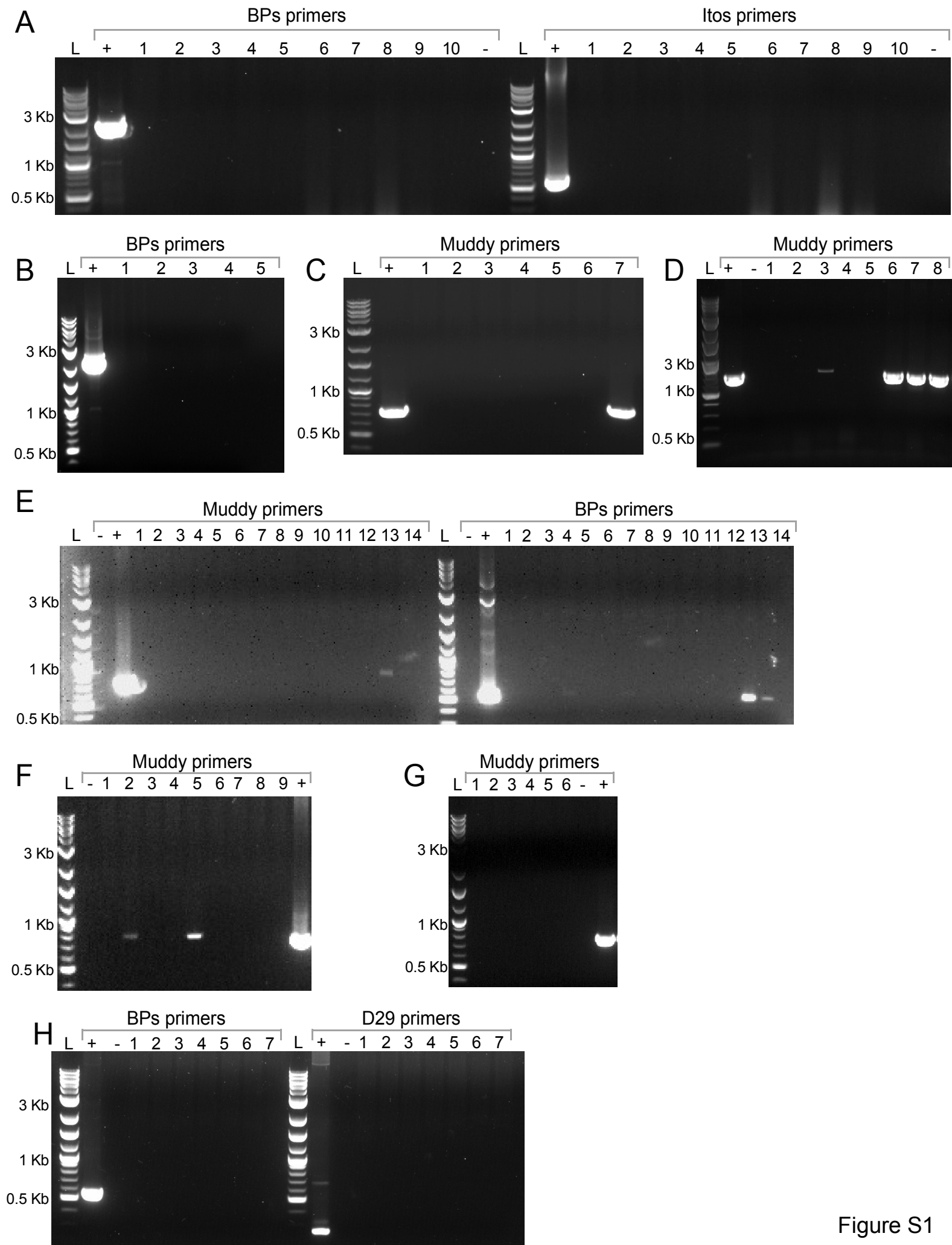

Figure S1

Supplement: ciac453_Supplementary_Data [file ciac453_supplementary_data.zip › FigS1_5-18-22 RMD.pdf]
